# Characterization of a Novel RNA Virus Discovered in the Autumnal Moth *Epirrita autumnata* in Sweden

**DOI:** 10.3390/v9080214

**Published:** 2017-08-08

**Authors:** Joachim R. de Miranda, Harald Hedman, Piero Onorati, Jörg Stephan, Olof Karlberg, Helena Bylund, Olle Terenius

**Affiliations:** 1 Department of Ecology, Swedish University of Agricultural Sciences, 750-07 Uppsala, Sweden; harald.hedman@gmail.com (H.H.); piero.onorati1@gmail.com (P.O.); jorg.stephan@slu.se (J.S.); helena.bylund@slu.se (H.B.); olle.terenius@slu.se (O.T.); 2 Department of Medical Sciences, Uppsala University, 751-85 Uppsala, Sweden; olof.karlberg@perkinelmer.com

**Keywords:** *Epirrita autumnata*, autumnal moth, Abisko virus, negevirus, *Operophtera brumata*, outbreak mortality

## Abstract

A novel, 10 kb RNA virus—tentatively named ‘Abisko virus’—was discovered in the transcriptome data of a diseased autumnal moth (*Epirrita autumnata*) larva, as part of a search for the possible causes of the cyclical nature and mortality associated with geometrid moth dynamics and outbreaks in northern Fennoscandia. Abisko virus has a genome organization similar to that of the insect-infecting negeviruses, but phylogenetic and compositional bias analyses also reveal strong affiliations with plant-infecting viruses, such that both the primary host origin and taxonomic identity of the virus remain in doubt. In an extensive set of larval, pupal, and adult autumnal moth and winter moth (*Operophtera brumata*) outbreak samples, the virus was only detected in a few adult *E. autumnata* moths as well as the single larval transcriptome. The Abisko virus is therefore unlikely to be a factor in the Fennoscandia geometrid population dynamics.

## 1. Introduction

Geometrid moth larvae are major defoliators of deciduous forests in central and northern Europe. In northern Fennoscandia, vast areas of mountain birch forests are recurrently defoliated by larvae of two geometrid species. In colder, continental areas the more cold-tolerant species, the autumnal moth (*Epirrita autumnata*) dominates while the winter moth (*Operophtera brumata*) prefers the warmer inland and coastal areas. However, the winter moth has started to appear at outbreak densities in locations where the autumnal moth was previously the sole outbreak species, probably due to climate change [1,2]. Both species show cyclical population dynamics with regular density fluctuations at cycles of 9–11 years, although the highest seasonal density at any one location and year does not always also correspond to an outbreak density. Winter moth populations show a delayed synchrony, with density peaks lagging one or two years behind the peak densities of the autumnal moth [3]. The cyclic pattern is partly driven by hymenopteran parasitoids attacking larvae and pupae [4]. The major cause of mortality at outbreak densities seems to be starvation. However, occasionally diseased autumnal moth larvae have been observed at outbreak density locations, while no diseased winter moth larvae were observed. When field-collected larvae are reared individually in the lab, only autumnal moth larvae develop disease symptoms and none of the winter moth larvae. Samples of similarly diseased autumnal moth larvae from an outbreak in northern Sweden during the 1960s were found to carry cytoplasmic polyhedrosis virus [5].

As part of an initiative to identify the possible causes of the lagged population dynamics synchrony between the two moth species, as well as possible causes for their elevated and differential mortality at outbreak densities, we screened the RNA and DNA from diseased *E. autumnata* larvae for the presence of pathogens. One of these larvae had a high abundance of a novel RNA virus, broadly related to the newly described negevirus family [6,7]. In this manuscript, we describe some of the characteristics of this virus.

## 2. Materials and Methods 

### 2.1. Sample Origins and RNA Purification

During the 2012–2013 geometrid outbreak in northern Fennoscandia, centered around Abisko and Kilpisjärvi (Supplementary Materials Figure S1), diseased *E. autumnata* larvae (Supplementary Materials Figure S2) were observed in Abisko. A total of 60 *E. autumnata* larvae (diseased and healthy), 86 pupae and 77 adults were collected for analysis, as well as 53 *O. brumata* pupae, from the six collection sites (Table 1). RNA and DNA was extracted from each individual insect using the All-Prep kit (Qiagen). Approximately 3 μg RNA was converted to cDNA with the GoScript cDNA synthesis kit (Promega) using a 50/50 mix of random hexamer and oligo-dT primers, diluted five-fold with water and stored at −20 °C until further use.

### 2.2. Sequencing and Genome Assembly

**Figure 1:**
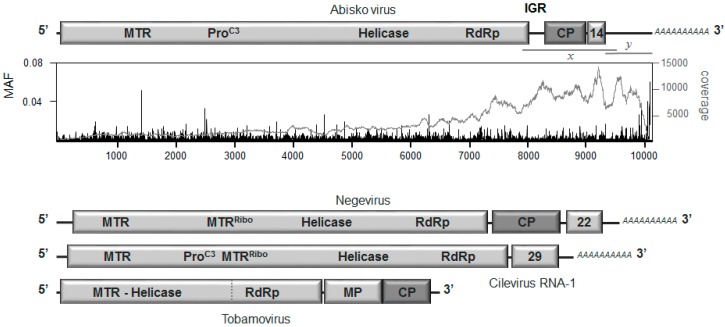
Organization of the putative Abisko virus genome, relative to that of the negeviruses, ciliviruses and tobamoviruses. Shown are the two major viral open reading frames (ORFs), the conserved domains identifying a methyl transferase gene (MTR), a C3 protease (C3-Pro), the helicase (Helicase), and the RNA-dependent RNA polymerase (RdRp) in ORF-1; an intergenic region (IGR); the virus capsid protein (CP), and an unidentified 14 kDa protein in ORF-2; the flanking 5′ and 3′ untranslated regions; the natural poly-A tail. Also shown are the unknown 22 kDa and 29 kDa proteins for the negeviruses and the cilevirus RNA-1, as well as the movement protein (MP) for the tobamoviruses. The graph shows the distribution of the Minor Allele Frequency (MAF) at each nucleotide position with respect to the consensus sequence (black bars), as well as the sequence coverage across the genome (grey line). The major junctions of the assembled sequence were confirmed by Sanger sequencing of specific reverse transcription quantitative polymerase chain reaction (RT-qPCR) fragments (*x*, *y*).

One microgram each of total RNA of two highly diseased *E. autumnata* larvae was sequenced by the SNP and SEQ platform of the SciLifeLab facility in Uppsala, Sweden using the default Illumina HiSeq technology (San Diego, CA, USA). The Abisko virus sequence was assembled from around 0.5 million end-paired ca. 100 nt sequences using the Trinity transcriptome assembly software [8], after filtering out low quality reads and removing the adapter sequences. The consensus sequence, coverage data, and variability profile were created using mpileup from Samtools v.0.1.8 (www.htslib.org, [9]) and an in-house script. The assembled Illumina sequence was confirmed through chain-termination sequencing [10] of uncloned Reverse Transcription-PCR fragments produced by primers EaNV-F8005 and EaNV-B9465 (Supplementary Materials Table S1), spanning the major junctions of the genome (Figure 1). The 3′ terminus was obtained by 3′RACE (Rapid Amplification of cDNA Ends), through priming the natural poly-A tail of the virus with anchored oligo-dT (V[T]^16^) for cDNA synthesis, followed by PCR with the EaNV-3RACE-F2 virus-specific forward primer (Supplementary Materials Table S1), using the FirstChoice RLM-RACE Kit (Applied Biosystems, Foster City, CA, USA). The identity of the reverse transcription quantitative polymerase chain reaction (RT-qPCR) fragments from the prevalence survey was also confirmed through sequencing. These PCR products were sequenced by Macrogen (Seoul, South Korea).

### 2.3. Virus Quantification

Quantitative qPCR was performed on a CFX Connect Real-Time PCR machine (BioRad, Hercules, CA, USA) using SSo Fast EvaGreen Supermix (Bio-Rad). The reaction volume was 20 μL containing 1 μL of the diluted cDNA template and 0.3 µM each of the forward and reverse primers (Supplementary Materials Table S1). Cycling conditions were 95 °C for 10 min, followed by 40 cycles at 95 °C for 15 s, 58 °C for 30 s, and 72 °C for 30 s. A melting curve (MC) analysis was included in each qPCR run confirm the identity of the PCR product. All assays were run in duplicate for each sample. The amount of virus RNA in each reaction was estimated by the CFX software through a calibration curve derived from a 10-fold dilution series of a cloned Abisko virus fragment of known concentration [11,12]. These amounts were multiplied by the various dilution factors from processing the samples to estimate the number of virus genome copies per insect.

### 2.4. Positive and Negative Strand RNA Quantification

The Abiskovirus genomic (positive) and replicative (negative) RNA strands were quantified separately using four pairs of strand-specific assays; two located in open reading frame 1 (ORF-1) and two located in ORF-2/3. Strand-specific cDNA was generated in 10 μL reactions containing 1 μL RNA plus 2 μM tagged cDNA primer (Supplementary Materials Table S1) heated to 70 °C for 5 min and cooled on ice; 20u MuMLV reverse transcriptase (Thermo Fisher K1612, Waltham, MA, USA), 10u RiboLock RNAse inhibitor (Thermo Fischer EO03819), 1 mM dNTP plus the MuMLV 5x reaction buffer. For each sample, a no-template, a no-primer, and a no-reverse transcriptase reaction were included in its suite of cDNA reactions, as essential cDNA negative controls [11,13]. Also included was a full suite of cDNA reactions for a virus-negative adult *E. autumnata* sample (25A), as a biological negative control. The cDNA reactions were incubated 60 min at 37 °C followed by 10 min at 70 °C. Excess cDNA primer was removed by adding 1u Exonuclease-I (Thermo Fischer 70073Z2500UN), incubating 30 min at 37 °C followed by 15 min at 70 °C, followed by diluting the cDNA 10-fold in sterile water [11,13]. The cDNAs were quantified by qPCR with the CFX Connect Real-Time PCR machine (BioRad) and the SSo Fast EvaGreen Supermix, using the tag to the cDNA primer and a virus-specific primer (Supplementary Materials Table S1) to ensure exclusive amplification of cDNA generated only with the tagged primer. The reaction volume was 20 μL containing 1 μL of the diluted cDNA template and 0.3 µM each of the forward and reverse primers. Cycling conditions were 95 °C for 10 min, followed by 35 cycles at 95 °C for 15 s, 58 °C for 30 s and 72 °C for 5 s, followed by a Melting Curve analysis to verify the identity of the PCR products. The no-primer and no-RT cDNA reactions were amplified with an equimolar mix of tag primer and a pool of all virus-specific primers, so as to enable the amplification of all possible illegitimate products. All reactions were run in triplicate, on separate plates. The Melting Curve data was used to identify positive amplifications, after which the mean and standard deviation of the corresponding quantification cycle (Cq) values were calculated and converted to estimated copies of cDNA per reaction, ±standard deviation, through external calibration curves established from 10-fold dilution series of the purified and quantified PCR products, covering four orders of magnitude dynamic range. The no-template cDNA controls and no-template qPCR controls were negative for all assays.

### 2.5. Phylogenetic Analyses

The predicted Abisko virus ORF1 and ORF2 amino acid sequences were queried with BLAST-P using default settings against the GenBank repository of non-redundant translated nucleotide sequences (nr database) at www.ncbi.nlm.nih.gov, identifying key viral amino acid motifs and a close similarity with negeviruses, ciliviruses and members of the *Virgaviridae*. For each unique virus taxon identified by BLAST-P, a single accession (Supplementary Materials Table S2) was extracted for multiple amino acid sequence alignment using COBALT [14]. The phylogenetic relationships between the viruses were inferred from this alignment using the Maximum Likelihood method based on the JTT matrix-based model [15], as implemented by MEGA6 [16]. All positions containing gaps and missing data were eliminated, resulting in 891 characters for analysis. Initial tree(s) for the heuristic search were obtained automatically by applying Neighbor-Join and BioNJ algorithms to a matrix of pairwise distances estimated using a JTT model, and then selecting the topology with superior log likelihood value. Statistical support for each node was determined by bootstrap analysis involving 1000 replicates [17]. The Abisko virus consensus sequence has been deposited at GenBank under accession KY662294. The partial Abisko virus sequences of the PCR fragments from the positive adult samples have been deposited under accessions MF001122-MF001125.

### 2.6. Compositional Bias Analyses

Virus genomes often mimic the compositional biases of their hosts’ genomes [18], such that they can be clearly separated by compositional bias according to their host type [19]. Compositional bias analyses can therefore assist in virus host identification. The codon usage and dinucleotide frequencies for the different codon regions of the virus nucleotide sequences (Supplementary Materials Table S2) were obtained at www.bioinformatics.org using the Sequence Manipulation Suite [20]. The dinucleotide frequency bias for each of the 16 dinucleotides was calculated as the ratio of the observed and expected dinucleotide frequencies, the latter being the product of the corresponding mononucleotide frequencies [19]. These multivariate data were used in correspondence analyses [19,21] to identify clusters of sequences with similar compositional bias in a multidimensional space. The analyses were conducted in R (www.R-project.org) using the ade4 package [22]. The translation initiation contexts between the −3 and +6 positions around the AUG start codons [23] of the different ORFs of the different viruses were tabulated by hand, converted to frequencies, and represented as stacked letters.

## 3. Results

### 3.1. Discovery

The starting point of the current study was the 2013 autumnal moth outbreak in northern Fennoscandia. Among the samples collected at the Abisko sampling site were a number of larvae with obvious disease symptoms (Supplementary Materials Figure S2). The RNA and DNA from two of the most affected larvae were submitted to Illumina sequencing. These sequences were assembled and compared to the public databases, with particular emphasis on pathogen signatures. The analyses of the DNA phase identified a novel baculovirus at low abundance (to be described in a separate publication). Analysis of the RNA phase on the other hand revealed in one of the samples an overwhelming abundance of microbial sequences, with 88% of the reads belonging to a 0.8 kb contig with 91% amino acid identity to an *Acinetobacter baumannii* hypothetical protein (GenBank WP_071209019.1) and 7% of the reads to a single 10 kb RNA virus. Only 4% of the reads belonged to the *E. autumnata* transcriptome and the remaining reads were mostly of bacterial and fungal origin. Given its abundance, this 10 kb Abisko virus merited further investigation.

### 3.2. Genome Organization

The Abisko virus sequence comprises a single-stranded RNA genome of about 10 kb, with a natural 3’ poly-A tail a relatively large (782 nt) 3′ untranslated region (3′UTR), a short 5′UTR, and three major ORFs, separated by an intergenic region (IGR) of about 229 nt and a 3 nt stretch following the end of the putative coat protein (Figure 1). ORF-1 contains clear domain signatures for a Methyl Transferase (MTR), a cysteine-protease (ProC3), a helicase, and an RNA-dependent RNA polymerase domains (RdRp). ORF-2 contains a coat protein (CP) while ORF-3 contains a 14 kDa protein with no clear homologues in the databases. The size and overall organization of the genome is most similar to that of the negeviruses, an insect-specific virus family [6,7]. To confirm the integrity of the genome and its organization, a 1.5 kb fragment spanning ORF-1, the intergenic region, ORF-2 and ORF-3 was amplified and sequenced directly by Sanger sequencing, confirming all of the junctions and gaps. Usually with this type of genome organization, the second and third ORFs are either translated by an internal ribosome entry site (IRES) located in the intergenic region [24] or from a subgenomic RNA [25,26]. No conserved sequence motifs for subgenomic promoters or IRES structures could be found in the Abisko genome. Indirect clues that Abisko virus may use a subgenomic RNA translation strategy are: (1) the highly elevated coverage for the 3′ region of the genome; (2) the presence of a viral methyl transferase, responsible for producing CAP-like structures for stabilizing the 3′ ends of viral (sub)genomic RNAs prior to translation through ‘normal’ ribosome AUG scanning; (3) most genera within the super-family to which Abisko virus and its close relatives belong tend to translate their 3′ ORFs through subgenomic RNAs [25] (but see [27]).

### 3.3. Replication and Translation

**Figure 2:**
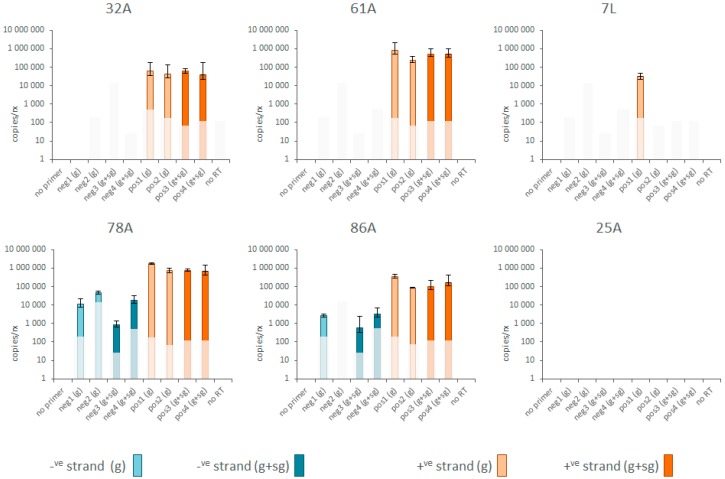
Amounts of negative-strand (blue bars) and positive-strand (orange bars) Abisko virus RNA in different *Epirrita autumnata* field-samples, as determined by four matching pairs of strand-specific RT-qPCR assays: negative-strand assays 1 to 4 (neg1~neg4) and its corresponding positive-strand assays 1 to 4 (pos1~pos4). The data refer to estimated copies cDNA per qPCR reaction. The lighter colored bars in each series refer to the assays located in ORF-1 (genomic region; (g)) while the darker colored bars refer to assays located in ORF-2 and ORF-3 (genomic + sub-genomic region; (g + sg)). The dimmed areas mark the detection thresholds for the assays at Cq = 35. The error bars mark the standard deviation for the assay based on triplicate runs. Samples 32A, 61A, 78A, and 86A are virus-positive adult *E. autumnata*; sample 7L is the original virus-positive *E. autumnata* larva; sample 25A is a virus-negative adult *E. autumnata*. The no-template cDNA controls and no-template qPCR controls were negative for all assays.

The infection status of Abiskovirus was assessed from evidence for negative-strand virus RNA and/or sub-genomic RNA in virus-positive samples. Both types of molecules are only produced during active infection and are therefore indicative that the virus is truly infectious in the host. However, only positive evidence for the presence of such molecules is informative, since these molecules are temporary infection intermediates and the infection process may already have passed the stage where such molecules are produced. The evidence was provided by four pairs of strand-specific RT-qPCR assays: two in the genomic region and two in the putative sub-genomic region (Figure 1; Supplementary Materials Table S1). Negative-strand Abisko virus RNA was detected by all four negative-strand assays in one of the four virus-positive *E. autumnata* adult samples (78A) and by three of the assays in another adult (86A), with assay neg2(g) just missing the detection threshold (Figure 2). No evidence of negative-strand RNA was recovered from the other two adult samples (31A; 61A) or the diseased larval sample (7L), even though positive-strand RNA was detected in abundance. The average ratio of positive to negative strand RNA for all pairs of assays was around 275:1 and 100:1 for samples 78A and 86A respectively, which is normal for RNA viruses [28]. There was no evidence of significant amounts of subgenomic RNA in any of the samples, since the assays located in ORF-1 (genomic RNA only) detected the same amount of RNA as those located in ORF-2 and ORF-3 (genomic plus subgenomic RNA: Figure 2; [29]). If Abisko virus indeed translates its ORFs through ribosome scanning, whether with or without subgenomic mRNAs, then the sequence context surrounding the AUG codon has a major role in deciding where translation is initiated [23]. This context is particularly important for ORF-3, which is downstream from ORF-2 with which it has to compete for translation initiation. The only ORF-3 AUG codon with a superior Kozak context than the putative start codon of ORF-2 is 91 nt downstream from the end of ORF-2 and without any product. The key positions of the Kozak sequence are at −3 (A/G), −1 (C/G), and +4 (G) relative to the AUG start codon [23]. The Kozak context of the nominal start of ORF-3 (UCU^8971^AUGUUA) is about as sub-optimal as can be and inferior to either of the possible ORF-2 start codons (UAA^8266^AUGCGC; AAU^8473^AUGACU), neither of which are particular optimal themselves. Therefore, unless ORF-3 is translated through a dedicated IRES hidden in the *CP* gene (e.g., [27]), it may well be produced as a read-through product of ORF-2, through a leaky UAG stop codon. Such a strategy has plenty of precedence elsewhere in the virus world, particularly for coat proteins that thus acquire extensions with potentially useful functions for cell entry, infectivity, and host-range [26].

### 3.4. Phylogenetic Relationships

**Figure 3:**
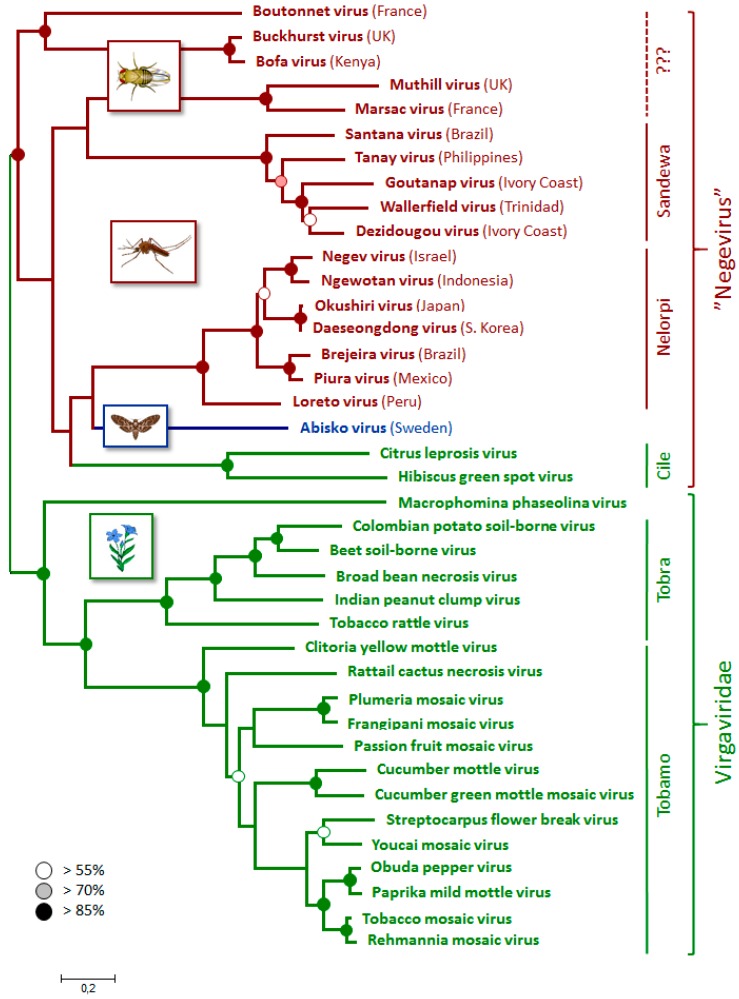
Phylogenetic relationships between the Abisko virus and related viruses, as inferred from 891 conserved amino acids in ORF-1 using Maxmim Likelihood criteria. The tree is drawn to scale, with branch lengths measured in the number of amino acid substitutions per site. Statistical support for the different nodes, shown by open, shaded, and solid circles, was determined by bootstrap analysis involving 1000 replicates. Shown next to the phylogram are the major genera and families to which different groups of viruses belong. The diagrams and branch coloring identify the principal host type of the different viruses: green = plants, red = insects, blue = Abiskovirus.

The phylogenetic analyses (Figure 3) confirm the broad association of the Abisko virus with the negeviruses, as implied by its genome organization, with wider affiliation to the plant-infecting *Virgaviridae* [7]. It lies in the middle of a poorly resolved section of the negeviruses together with the plant-infecting cileviruses, which have a quite different genome organization (Figure 1). The Abiskovirus RdRp and coat protein (ORF-2), are also closely related to their homologues in the *Virgaviridae*, which will have contributed to the uncertainty of its phylogenetic placement within the negeviridae. This is in itself not particularly unusual: RNA viruses in particular are well capable of acquiring gene cassettes from a variety of different origins, including related viruses [30] and even hosts [31]. However, it makes both the classification and likely host origin of the virus difficult to establish.

### 3.5. Incidence and Origin

The virus has a rather low prevalence in *E. autumnata*, with 0/86 pupae, 1/60 larvae, and 4/77 adults registering positive for the virus by RT-qPCR, as well as 0/52 *O. brumata* pupae (Table 1). The identity of the PCR fragments of the positive samples was confirmed by sequence analysis. The five positive samples were collected from three different sampling sites. The virus titers were about three orders of magnitude higher in the four virus-positive adult samples (2~25 × 10^9^ copies/individual) than in the larval sample (5 × 10^6^ copies/individual). These data, indicating host status for the adults but uncertainty for larvae or pupae, present an interesting conundrum concerning the origin and transmission of Abisko virus. Since the adult moths do not feed, are only alive for a few weeks in late autumn and the females are largely immobile [32], they represent dead-end hosts for the virus. Either the virus has a more significant host elsewhere and the infection of *E. autumnata* adults is an accidental irrelevance, possibly involving acquisition during the larval phase and undetectable persistence through the pupal stage. Alternatively, *E. autumnata* is a primary host and transmission between individuals is through a mobile vector (parasitic wasps, for example).

**Figure 4:**
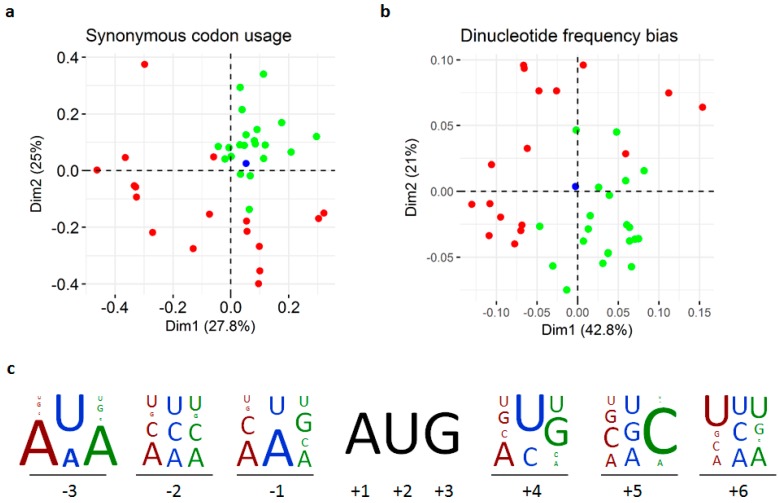
Results of the compositional bias and translation initiation context analyses (**a**) synonymous codon usage bias, (**b**) dinucleotide frequency bias, (**c**) translation initiation context. The green dots/letters represent plant-infecting viruses, the red dots/letters represent insect-infecting viruses and the blue dots/letters represent the Abisko virus.

Virus genomes often mimic the compositional biases of their hosts’ genomes [18], such that they can be clearly separated by compositional bias according to their host type [19]. Compositional bias analyses can therefore sometimes assist in virus host identification. However, although the compositional bias analyses confirm the broad compositional differences between insect- and plant-infecting viruses [19], none of them assign the Abisko virus convincingly to either group (Figure 4).

## 4. Discussion

In this manuscript, we describe the discovery and a limited set of molecular features of a novel 10 kb RNA virus that was assembled from transcriptome data of a diseased *E. autumnata* larva from the 2012–2013 geometrid outbreak in northern Fennoscandia. Subsequent screening of an extensive set of natural samples revealed that the virus could not be detected in either larvae (except for the original transcriptome sample) or pupae of both *E. autumnata* and *O. brumata*, and that it could be detected in only four adult *E. autumnata*, collected from three different sites. The dispersed distribution, generous titers, and clear evidence for virus replicative-strand intermediates indicate that adult *E. autumnata* are a true host of the virus, while the absence of the virus or its replication intermediates from all pupae and all but one larva suggests that these are not hosts. Since the adults live only a few weeks, are not very mobile, and do not feed or defecate, the persistence of Abisko virus in the adult autumnal moth population is difficult to explain unless other transmission routes or hosts are involved. The genome organization and phylogenetic analyses identify Abisko virus most closely with the negeviruses, a newly described group of insect-infecting viruses [6,7], but with sufficient uncertainty to leave both its classification and primary hosts status in doubt. Consequently, we decided to conservatively name the virus after the place it was found (Abisko), following the convention used for its closest relatives, the negeviruses, but leave its classification otherwise unassigned.

## Figures and Tables

**Table 1:** Incidence of Abisko virus in natural samples of larvae, pupae and adults collected during the 2012–2013 geometrid outbreak in northern Fennoscandia (Supplementary Materials Figure S1). The data refer to the number of samples and number of virus-positive samples (in brackets) at each site.

Location		Lat/Lon	*Epirrita autumnata*			*Operophtera brumata*		
			La	Pu	Ad	La	Pu	Ad
Nuorgam	Finland	70.09155, 27.89978	1 (0)	9 (0)	-	-	19 (0)	-
Utsjoki	Finland	69.87178, 27.21863	-	6 (0)	-	-	-	-
Kilpisjärvi	Finland	69.06390, 20.66048	-	18 (0)	23 (1)	-	-	-
Neiden	Norway	69.67379, 29.47426	-	12 (0)	-	-	22 (0)	-
Gratangen	Norway	68.72343, 17.59460	-	33 (0)	24 (2)	-	8 (0)	-
Abisko	Sweden	68.30850, 18.51059	59 (1)	8 (0)	30 (1)	-	3 (0)	-

## Supplementary Materials

The following are available online at www.mdpi.com/1999-4915/9/8/214/s1, Figure S1: Geographic origin of samples, Figure S2: Natural disease symptoms, Table S1: RT-qPCR assay details, Table S2: Accessions of related viruses.
