# Supplementary material for: Characterization of a Novel RNA Virus Discovered in the Autumnal Moth Epirrita autumnata in Sweden
Source: Viruses. 2017 Aug 8;9(8):214. doi: 10.3390/v9080214 (PMC5580471; doi:10.3390/v9080214)
Supplement: Supplementary file 1 [file viruses-09-00214-s001.pdf]

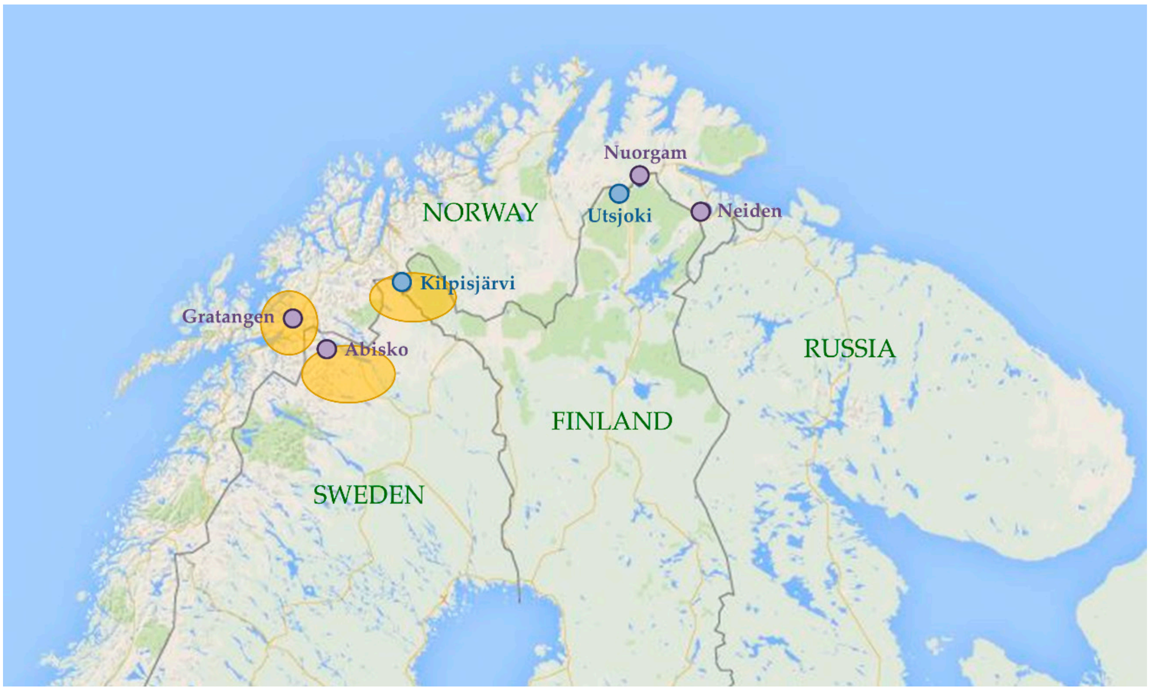

Figure S1. Sampling sites of the 2012–2013 geometrid outbreak in northern Fennoscandia. Sites where both *E. autumnata* and *O. brumata* were found are in purple, those where only *E. autumnata* was found in blue. Areas with high densities of geometrids, and likelihood of diseased individuals, are shaded in yellow.

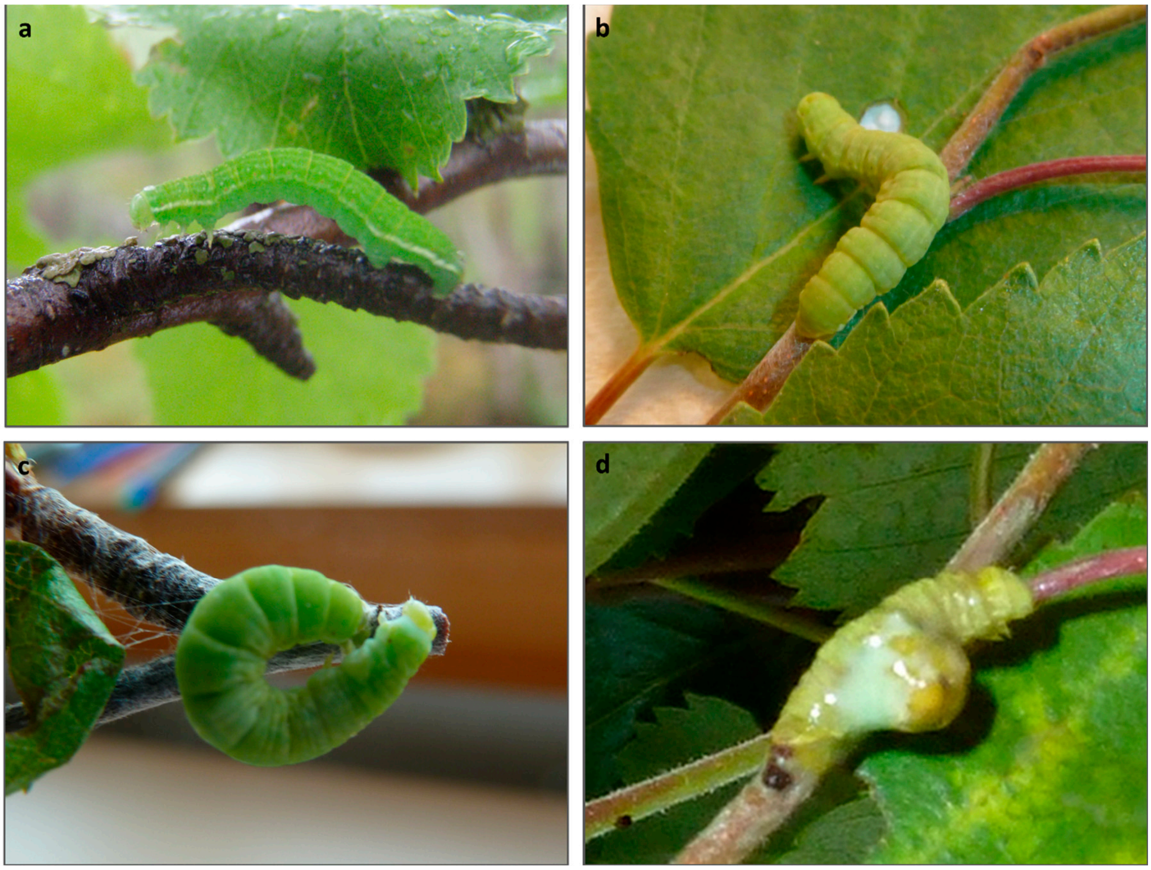

Figure S2. Images of disease progression in *E. autumnata* larvae collected near Abisko during 2012. (**a**) healthy larva (**b**) opaque discoloring (**c**) spinning and fastening (**d**) exploding larva.

Table S1. Details of the RT-PCR assays used in the study, including the purpose of the assay; the primer names and sequences; the PCR product size (nt); the Abiskovirus genome location of the assay (helicase, RNA-dependent RNA polymerase (RdRp), coat protein (CP), 14 kDa protein); the assay reaction efficiency (*E*); the log-linearity of the calibration curve (r^2); and the peak melting temperature of the qPCR product (T_m).

assay		primers	sequence (5′-3′)	size	region	*E*	r^2	T_m
sequencing	RT-PCR	EaNV-F8005<br>EaNV-R9465	GTATGGATCCGTCTATGG<br>CAGACATGGTTAGAGAAGG	1461	ORF1-ORF3	*n.a.*	*n.a.*	*n.a.*
		EaNV-3RACE-F2<br>Oligo-dT	AATTGCCATTACCAGCGTGGCGT<br>TTTTTTTTTTTTTGGTTTTTAAAC	851	3'-RACE	*n.a.*	*n.a.*	*n.a.*
titre	RT-qPCR	EaNV-F6572<br>EaNV-R6790	GCTACCCGGAAATTCTAC<br>CTCTTAATCATCGCTAACAG	219	RdRp	1.97	1.00	82.5 °C
negative strand	(-) cDNA	EaNV-NegF5419	agcctgcgcaccgtggCTTGTCTGGTCTTAACACC	200	helicase	1.91	1.00	79.0 °C
	qPCR	EaNV-Tag<br>EaNV-R5565	agcctgcgcaccgtgg<br>AACATGAGAGCTTCCTCGAC					
	(-) cDNA	EaNV-NegF6572	agcctgcgcaccgtggGCTACCCGGAAATTCTAC	235	RdRp	1.66	1.00	81.3 °C
	qPCR	EaNV-Tag<br>EaNV-R6790	agcctgcgcaccgtgg<br>CTCTTAATCATCGCTAACAG					
	(-) cDNA	EaNV-NegF8450	agcctgcgcaccgtggCCTTTGATTTCGTCGAATCC	181	CP	2.00	1.00	79.5 °C
	qPCR	EaNV-Tag<br>EaNV-R8578	agcctgcgcaccgtgg<br>CGGAATCCTGTGAGCAAC					
	(-) cDNA	EaNV-NegF9084	agcctgcgcaccgtggTTTGGAAGATTTCGCTTCC	228	14 kD	1.87	1.00	81.5 °C
	qPCR	EaNV-Tag<br>EaNV-R9259	agcctgcgcaccgtgg<br>GGAAGGTGTAACCAGTAAC					
positive strand	(+) cDNA	EaNV-PosR5565	agcctgcgcaccgtggAACATGAGAGCTTCCTCGAC	200	helicase	1.92	1.00	78.8 °C
	qPCR	EaNV-Tag<br>EaNV-F5419	agcctgcgcaccgtgg<br>CTTGTCTGGTCTTAACACC					
	(+) cDNA	EaNV-PosR6790	agcctgcgcaccgtggCTCTTAATCATCGCTAACAG	235	RdRp	1.99	1.00	80.6 °C
	qPCR	EaNV-Tag<br>EaNV-F6572	agcctgcgcaccgtgg<br>GCTACCCGGAAATTCTAC					
	(+) cDNA	EaNV-PosR8578	agcctgcgcaccgtggCGGAATCCTGTGAGCAAC	181	CP	1.95	1.00	80.0 °C
	qPCR	EaNV-Tag<br>EaNV-F8450	agcctgcgcaccgtgg<br>CCTTTGATTTCGTCGAATCC					
	(+) cDNA	EaNV-PosR9259	agcctgcgcaccgtggGGAAGGTGTAACCAGTAAC	228	14 kD	1.95	1.00	81.1 °C
	qPCR	EaNV-Tag<br>EaNV-F9084	agcctgcgcaccgtgg<br>TTTGGAAGATTTCGCTTCC					

Table S2. GenBank accession numbers, names, classification and principal hosts of the viruses used in the phylogenetic and compositional bias analyses.

Accession	Virus	Genus	Host
**KY662294**	**Abisko virus**	**?**	**Lepidoptera**
KU754539	Boutonnet virus	?	Diptera
KU754516	Buckhurst virus	?	Diptera
KU754515	Bofa virus	?	Diptera
KU754517	Muthill virus	?	Diptera
KU754518	Marsac virus	?	Diptera
JQ675606	Santana virus	Sandewavirus	Diptera
KF425262	Tanay virus	Sandewavirus	Diptera
KF588036	Goutanap virus	Sandewavirus	Diptera
KX518837	Wallerfield virus	Sandewavirus	Diptera
JQ675604	Dezidougou virus	Sandewavirus	Diptera
KX518776	Negev virus	Nelorpivirus	Diptera
KX518777	Ngewotan virus	Nelorpivirus	Diptera
AB972669	Okushiri virus	Nelorpivirus	Diptera
KU095841	Daeseongdong virus	Nelorpivirus	Diptera
KX518762	Brejeira virus	Nelorpivirus	Diptera
KX518786	Piura virus	Nelorpivirus	Diptera
JQ675611	Loreto virus	Nelorpivirus	Diptera
DQ388512	Citrus leprosis virus RNA-1	Cilevirus	Flowering plants
HQ852052	Hibiscus green spot virus RNA-1	Higrevirus	Flowering plants
KF537660	Macrophomina phaseolina virus	?	Flowering plants
KT225271	Colombian potato soil-borne virus RNA-1	Pomovirus	Flowering plants
FJ971717	Beet soil-borne virus RNA-1	Pomovirus	Flowering plants
D86636	Broad bean necrosis virus RNA-1	Pomovirus	Flowering plants
X99149	Indian peanut clump virus RNA-1	Pecluvirus	Flowering plants
X06172	Tobacco rattle virus RNA-1	Tobravirus	Flowering plants
JN566124	Clitoria yellow mottle virus	Tobamovirus	Flowering plants
JF729471	Rattail cactus necrosis virus	Tobamovirus	Flowering plants
KJ395757	Plumeria mosaic virus	Tobamovirus	Flowering plants
JN555602	Frangipani mosaic virus	Tobamovirus	Flowering plants
AB261167	Cucumber mottle virus	Tobamovirus	Flowering plants
D12505	Cucumber green mottle mosaic virus	Tobamovirus	Flowering plants
HQ38954	Passion fruit mosaic virus	Tobamovirus	Flowering plants
AM040955	Streptocarpus flower-break virus	Tobamovirus	Flowering plants
AY318866	Youcai mosaic virus	Tobamovirus	Flowering plants
D13438	Obuda pepper virus	Tobamovirus	Flowering plants
AB089381	Paprika mild mottle virus	Tobamovirus	Flowering plants
AB628188	Rehmannia mosaic virus	Tobamovirus	Flowering plants
EF392659	Tobacco mosaic virus	Tobamovirus	Flowering plants
